# Impact of the new membrane-targeting lipoglycopeptide antibiotic MCC5145 on the treatment of bacteremic pneumococcal pneumonia in mice

**DOI:** 10.1128/spectrum.04459-22

**Published:** 2023-08-22

**Authors:** Abiodun D. Ogunniyi, Hang Thi Nguyen, Karl A. Hansford, Matthew A. Cooper, Darren J. Trott, Mark A. T. Blaskovich

**Affiliations:** 1 Australian Centre for Antimicrobial Resistance Ecology, School of Animal and Veterinary Sciences, The University of Adelaide, Roseworthy, South Australia, Australia; 2 Centre for Superbug Solutions, Institute for Molecular Bioscience, The University of Queensland, Brisbane, Queensland, Australia; Hartford Hospital, Hartford, Connecticut, USA

**Keywords:** *Streptococcus pneumoniae*, pneumococcus, pneumonia, immunocompromised hosts, lipoglycopeptide, bioluminescence, antimicrobial agents, bacteremic pneumococcal pneumonia

## Abstract

Bacteremic *Streptococcus pneumoniae* pneumonia is one of the most severe forms of invasive pneumococcal disease (IPD) and with particularly high case-fatality rates among the elderly and individuals with comorbidities, exacerbated by rising antibiotic resistance and time to initiation of therapy. Here, we examined the efficacy of the preclinical “vancapticin” glycopeptide MCC5145 against fulminant infection by *S. pneumoniae* serotype 2 strain D39 in a bioluminescent, neutropenic mouse model of bacteremic pneumonia. MCC5145 is a semisynthetic vancomycin derivative chemically modified at the *C*-terminus with a membrane-targeting motif designed to preferentially bind the anionic bacterial surface. We show that similar to vancomycin, subcutaneous administration of MCC5145 to mice 1 day after intranasal infection with a bioluminescent derivative of *S. pneumoniae* D39 elicited time and concentration-dependent reduction in total flux in the lungs and blood. Together, our finding supports the further development of MCC5145 as a potential new treatment option for pneumonia and/or bacteremic pneumonia in clinical settings, particularly for immunocompromised individuals.

**IMPORTANCE:**

*S. pneumoniae* (the pneumococcus) causes severe community acquired lung and blood infection, especially among the elderly and people with underlying medical conditions and/or weakened immune systems. The rising incidence of antibiotic resistance and delays between diagnosis of infection and commencement of effective therapy make treatment difficult and result in high mortality rates. In this work, we show that a new derivative (MCC5145) of an existing antibiotic (vancomycin) rapidly eradicated lethal pneumococcal challenge from the lungs and blood of mice with a suppressed immune system. Our findings support that MCC5145 is a promising option for the treatment of lung and blood infections caused by the pneumococcus at point-of-care settings, particularly for the elderly and individuals with a weakened immune system.

## OBSERVATION

*Streptococcus pneumoniae* (the pneumococcus) continues to be the leading cause of lower respiratory tract infections despite the availability of vaccines and antibiotics, accounting for half a million pneumonia-related deaths in children worldwide, particularly in developing countries (1). In developed countries, deaths from pneumococcal disease occur primarily among the elderly (≥65 yr of age), with case fatality rates of 10–20% for pneumonia (2) and up to 40% for bacteremic pneumococcal pneumonia (3, 4). Management of invasive pneumococcal disease is being hindered by the shortcomings of pneumococcal conjugate vaccines, namely cost, immunogenicity, limited serotype coverage, and replacement disease caused by nonvaccine serotypes (5, 6). Additionally, the most recent pneumococcal isolates exhibit reduced susceptibility to antibiotics commonly used to treat life-threatening IPD, including third-generation cephalosporins, fluoroquinolones, and carbapenems (7, 8). The use of daptomycin to treat pneumococcal pneumonia is also not recommended due to its inactivation by lung surfactants (9).

**Fig 1:**
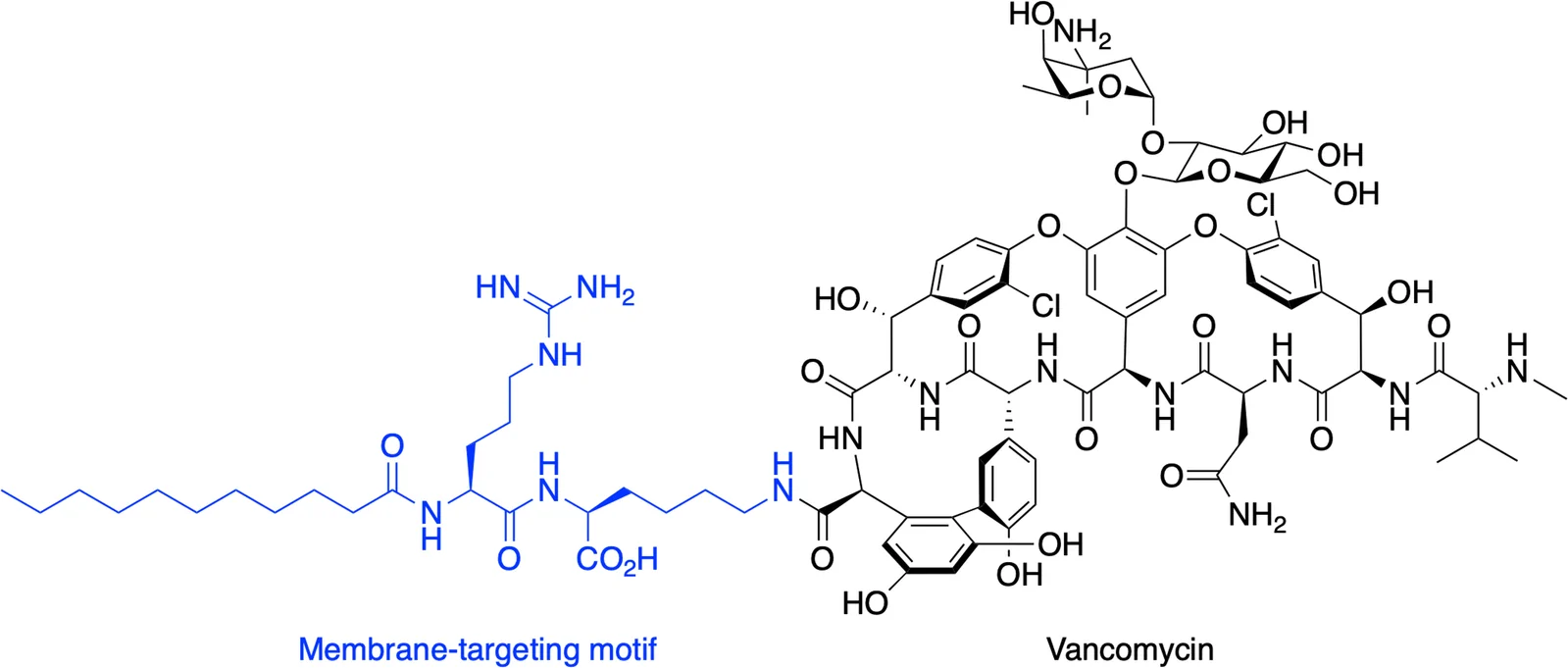
The structure of MCC5145.

We recently reported on the development of MCC5145 (a semisynthetic vancomycin derivative) as a promising new lipoglycopeptide antibiotic with clinical potential against a wide range of Gram-positive pathogens (10). Our studies included extensive *in vitro* characterization, subcutaneous and intravenous (IV) pharmacokinetics in mice and rats, extensive screening for drug-like properties, mutagenicity and genotoxicity, off-target screening, CYP inhibition/induction, and cardiac liabilities. We demonstrated the effectiveness of MCC5145 in multiple *in vivo* infection models, including those with difficult-to-eradicate biofilms. We also demonstrated reduced *in vivo* nephrotoxicity for MCC5145 compared to vancomycin following a 7 day repeat dose (40 mg/kg/day IV) toxicology study in mice at exposure levels commensurate with efficacy against *S. pneumoniae* bacteremia. MCC5145 appends a membrane binding motif to the *C*-terminus of the vancomycin core, using a lysine linker to attach a single arginine residue capped with undecanoic acid (Fig. 1). Within this construct, the hydrophobic tail is designed to insert into the bacterial membrane, whereas the positively charged arginine assists to bring the drug to the negatively charged bacterial surface, providing additional bacterial interactions to supplement the Lipid II binding of the vancomycin core.

Based on these promising results, we sought to determine the efficacy of MCC5145 at eradicating lethal *S. pneumoniae* from the lungs and blood of mice in a neutropenic intranasal (IN) model of bacteremic pneumococcal pneumonia using a bioluminescent derivative of a virulent serotype 2 *S*. *pneumoniae* strain D39, herein referred to as D39LUX (11, 12). The MICs of MCC5145, vancomycin, and daptomycin vs D39LUX in the absence of surfactant are 0.016, 0.5, and 0.5 µg/mL, respectively, similar to values obtained for these antibiotics against *S. pneumoniae* ATCC 700677. Our previous *in vitro* studies showed reduced activity for MCC5145 against *S. pneumoniae* ATCC 700677 in the presence of an artificial lung surfactant (5% Survanta). However, MICs were still below or at 1 µg/mL for MCC5145 and vancomycin, in contrast to the substantial reduction in activity observed for daptomycin in surfactant (10).

The study was conducted in accordance with the requirements of the Australian Code of Practice for the Care and Use of Animals for Scientific Purposes (8th Edition 2013) and the South Australian Animal Welfare Act (1985) and was reviewed and approved by the Animal Ethics Committee of the University of Adelaide (Approval Number S-2015-151). For this assay, outbred male Swiss (CD1) mice, 5–6 weeks (approx. 25–32 g, 6–7 mice per group) were rendered neutropenic by cyclophosphamide administered intraperitoneally (IP) at 150 and 100 mg/kg body weight in 0.1 mL volumes, 4 and 1 day(s), respectively, prior to infection (13). On the day of infection, mouse-passaged D39LUX was grown without agitation in serum broth (nutrient broth containing 10% (vol/vol) heat-inactivated horse serum) to *A*_600 nm_ = 0.16 (equivalent to 5 × 10^7^ CFU per mL) as described previously (14), centrifuged at 4,000 × *g* for 5 min, washed twice in phosphate-buffered saline (PBS), and resuspended in the same buffer at a concentration of 2 × 10^8^ CFU per mL. For infection, mice were anesthetized by IP injection of pentobarbital sodium (Pentobarbital sodium; Ilium) at a dose of 66 mg/kg body weight and challenged IN with 50 µL of the D39LUX suspension in PBS containing approximately 1 × 10^7^ CFU.

Throughout the experiment, all mice were monitored and scored daily for weight, coat condition, posture, behavior, hydration, bleeding, diarrhoea, breathing, and movement and the clinical conditions recorded in the Clinical Record Sheet approved by the University of Adelaide Ethics Committee. All criteria were scored as present (1) or absent (0), except for reluctance to move, which was scored out of 3. The cut-off point was a total score of 4 or weight loss >20%.

On day 1 post-infection, mice were imaged in both ventral and dorsal positions on the IVIS Lumina XRMS Series III system (Caliper LifeSciences). Immediately thereafter, mice in group 1 were treated once subcutaneously (SC) with 100 µL sodium acetate buffer (vehicle-only control), and mice in groups 2 and 3 were treated with MCC5145 at 2 mg/kg SC or 20 mg/kg SC, respectively, while mice in group 4 received vancomycin at 20 mg/kg SC. The MCC5145 solutions were prepared in acetate buffered pH 5.5 5% glucose, using 5 mM acetic acid and 45 mM sodium acetate in 5% glucose. Dose selection was guided by the consideration that the MIC potency of MCC5145 is attenuated in the presence of 5% lung surfactant, and our previous observation that MCC5145 could rescue mice from a lethal challenge of D39LUX in an intraperitoneal sepsis model as low as 2 mg/kg SC at 2- and 6 h post-infection (10). Accordingly, we increased the dose of MCC5145 10-fold (i.e., 20 mg/kg) as a reasonable starting point, using the same dose of vancomycin for direct comparison. The clinical conditions of all mice were recorded on the Clinical Record Sheet approved by the University of Adelaide Animal Ethics Committee.

At days 2 and 3 post-infection, all surviving mice in each group were imaged and treated as described above. Mice were further monitored for signs of distress and those that had become moribund or showed any evidence of distress were humanely killed by cervical dislocation. At day 4 post-infection, surviving mice were further subjected to bioluminescence imaging and clinical conditions were recorded, at which time the experiment was terminated. In all experiments, signals were collected from a defined region of interest and total flux intensities (photons/s) were analyzed using Living Image Software 4.7.2. Differences in median survival times (time to moribund) for mice between groups were analyzed by the log-rank (Mantel-Cox) tests. Differences in luminescence signals between control and treated groups were compared by unpaired *t*-tests (two-tailed). In the current manuscript, lung homogenates and/or blood were not taken for quantitative culture, based on our large body of evidence from previous published work with D39LUX (10 – 12), which consistently showed a strong correlation between quantitative culture and luminescence signals.

**Fig 2:**
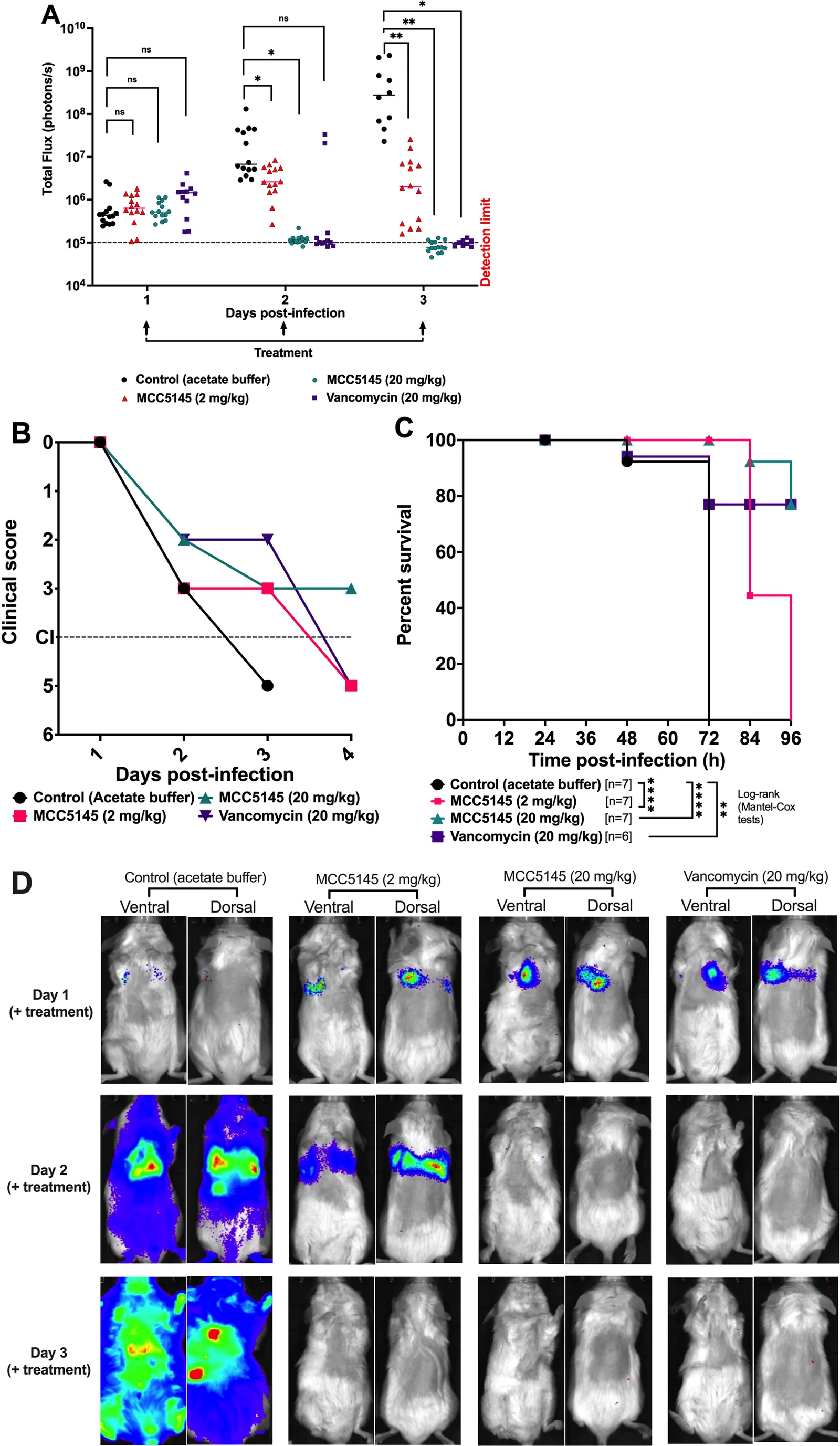
*In vivo* bioluminescent bacteremic pneumococcal pneumonia model. Outbred male Swiss (CD1) mice, 5–6 weeks old (approx. 25–32 g); 6–7 mice per group were rendered neutropenic and then challenged IN with 1 × 10^7^ CFU bioluminescent *S. pneumoniae* D39 (D39LUX) under anesthesia. (**A**) Combined (ventral and dorsal) luminescence signal comparisons between control (untreated) and drug-treated mice over time. ns, not significant; *, *P* < 0.05; **, *P* < 0.01; unpaired *t*-test (two-tailed); (**B**) Median clinical score comparisons between groups; CI = Clinical index, set at a threshold of 4, based on an aggregate score of the clinical conditions of mice recorded on a Clinical Record Sheet. Beyond this point, mice were considered moribund and humanely killed; (**C**) Survival analysis for control (untreated) and mice treated with the indicated drugs. **, *P* < 0.01; ****, *P* < 0.0001; Log-rank (Mantel-Cox test); (**D**) Representative ventral and dorsal images of CD1 mice challenged with D39LUX. Mice were treated with MCC5145 at 2 mg/kg or 20 mg/kg, vancomycin at 20 mg/kg or control (vehicle only) at day 1, day 2, and day 3 post-infection. Mice were imaged on the IVIS Lumina XRMS Series III system at the indicated times.

Results showed that after the first treatment with 20 mg/kg of MCC5145, there was a marked reduction in total flux compared to the total flux in mice treated with 2 mg/kg MCC5145 (Fig. 2A), with one mouse in the vancomycin group showing little response. After a second treatment, there was a complete loss of luminescence signals from mice treated with 20 mg/kg MCC5145 or vancomycin at 20 mg/kg, but there was residual luminescence from mice treated with 2 mg/kg MCC5145. However, luminescence signals increased exponentially in control (untreated) mice over the duration of the experiment concomitant with higher clinical scores (Fig. 2B), and all mice in this group had become moribund between days 2 and 3 post-infection (Fig. 2C and D), a classical disease pathogenesis pattern for mice infected IN with D39 (15). We observed a decreased time needed to reduce total flux in the lungs and blood for MCC5145 at 20 mg/kg compared to vancomycin at the same dose (although overall survival rates at 96 h were the same, Fig. 2C), compatible with the observed fourfold lower *in vitro* MIC value for MCC5145 for *S. pneumoniae* in the presence of lung surfactant and the extended mouse pharmacokinetic profile of MCC5145 compared to vancomycin (10).

The similar time and concentration dependent reduction in total flux of pneumococci compared to vancomycin at exposure levels commensurate with efficacy against *S. pneumoniae* bacteremia confirms that preclinical candidate MCC5145 has potential as a new treatment option for pneumococcal pneumonia and/or bacteremic pneumococcal pneumonia. This is particularly of relevance for the treatment of immunosuppressed individuals and critically ill patients at point-of-care settings. It is important to note that the results presented in this study are limited to the use of one bacterial strain in one experiment. Future studies would necessarily include testing of a broader set of strains to confirm uniformity and reproducibility.
